# Heterogeneity revealed through meta-analysis might link geographical differences with nasopharyngeal carcinoma incidence in Han Chinese populations

**DOI:** 10.1186/s12885-015-1607-0

**Published:** 2015-08-26

**Authors:** Wen-Hui Su, Chi-Cking Chiu, Yin Yao Shugart

**Affiliations:** 1 Department of Biomedical Sciences, Graduate Institute of Biomedical Sciences, College of Medicine, Chang Gung University, Taoyuan, Taiwan; 2 Chang Gung Molecular Medicine Research Center, Chang Gung University, Taoyuan, Taiwan; 3 Division of Intramural Research Programs, Unit on Statistical Genomics, National Institute of Mental Health, Bethesda, MD USA; 4 Department of Gastroenterology, Johns Hopkins Medical School, Baltimore, MD USA

## Abstract

**Background:**

Nasopharyngeal carcinoma (NPC) is an epithelial malignancy highly prevalent in southern China, and incidence rates among Han Chinese people vary according to geographic region. Recently, three independent genome-wide association studies (GWASs) confirmed that *HLA-A* is the main risk gene for NPC. However, the results of studies conducted in regions with dissimilar incidence rates contradicted the claims that *HLA-A* is the sole risk gene and that the association of rs29232 is independent of the *HLA-A* effect in the chromosome 6p21.3 region.

**Methods:**

We performed a meta-analysis, selecting five single-nucleotide polymorphisms (SNPs) in chromosome 6p21.3 mapped in three published GWASs and four case–control studies. The studies involved 8994 patients with NPC and 11,157 healthy controls, all of whom were Han Chinese.

**Results:**

The rs2517713 SNP located downstream of HLA-A was significantly associated with NPC (*P* = 1.08 × 10^−91^, odds ratio [OR] = 0.58, 95 % confidence interval [CI] = 0.55–0.61). The rs29232 SNP exhibited a moderate level of heterogeneity (I^2^ = 47 %) that disappeared (I^2^ = 0 %) after stratification by moderate- and high-incidence NPC regions.

**Conclusions:**

Our results suggested that the *HLA-A* gene is strongly associated with NPC risk. In addition, the heterogeneity revealed by the meta-analysis of rs29232 might be associated with regional differences in NPC incidence among Han Chinese people. The higher OR of rs29232 and the fact that rs29232 was independent of the *HLA-A* effect in the moderate-incidence population suggested that rs29232 might have greater relevance to NPC incidence in a moderate-incidence population than in a high-incidence population.

**Electronic supplementary material:**

The online version of this article (doi:10.1186/s12885-015-1607-0) contains supplementary material, which is available to authorized users.

## Background

Nasopharyngeal carcinoma (NPC), a malignancy that forms in the epithelium of the nasopharynx, has a distinct geographic distribution and is highly prevalent in southern China, Southeast Asia, and North Africa. Although all Han Chinese populations exhibit an increased risk of NPC, the incidence rate varies by region. For example, male populations in Guangdong and Guangxi in southern China have consistently exhibited a higher incidence rate (20.6–39.94/100 000 person-years) compared with those in moderate-incidence regions, such as Taiwan (8.6/100 000 person-years), and those in most of the Western world (less than 1/100 000 person-years) [1–5]. The etiology of NPC is multifactorial, involving genetic components, Epstein–Barr virus infection, and other types of environmental exposure [1]. The variations in NPC incidence might be due to differences in environmental exposure among geographic regions; however, the genetic components underlying the differences in incidence in Han Chinese populations remain underexplored.

The genetic association of human leukocyte antigen (HLA) class I genes, particularly *HLA-A*, with NPC was established in 1974 [6] and has been confirmed in more than 100 association studies adopting traditional HLA genotyping techniques. Studies have consistently identified an association between NPC and HLA-A*1101, HLA-A*0207, and HLA-B*5801 [7–9]. The distribution of these three alleles in the human genome appears to be consistent with the geographical distribution of NPC incidence in southeastern China; the allele frequency is particularly high in regions with high NPC incidence rates [10–12]. However, no difference in HLA allele frequency has been observed in the results of NPC association studies conducted in regions with various incidence rates [13–15], suggesting that the HLA genes might not directly lead to differences in NPC incidence.

**Fig. 1:**
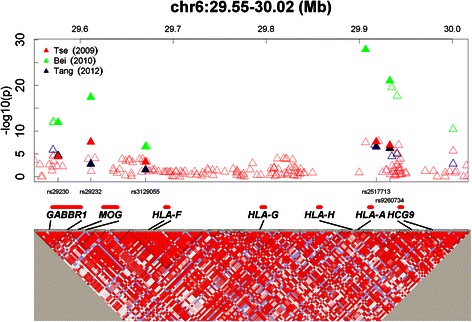
Chromosome 6p21.3 polymorphisms discovered in three NPC GWASs. **Top**: The triangles indicate the *P* values reported by the GWASs on a negative logarithmic scale according to the chromosome locations of the SNPs. **Red**: Tse [16]; **Green**: Bei [17]; **Blue**: Tang [18]. The solid triangles indicate the SNPs used in the meta-analysis. The hollow triangles indicate the other SNPs listed in the GWASs. **Bottom**: Detailed LD structure depicted in HaploView by using control samples from the NPC GWAS in Taiwan [16]. The increasing intensities of red represent lower D’ values

Three independent genome-wide association studies (GWASs) [16–18] have identified multiple significant association signals in chromosome 6p21.3 near the *HLA-A* gene, which exhibited extremely strong linkage disequilibrium (LD) (Fig. 1). These observations raised the question as to whether these associated single-nucleotide polymorphisms (SNPs) represent an independent effect or are only proxies of the *HLA-A* gene. Studies conducted in medium- and high-incidence regions (Taiwan [16, 19], and Guangdong and Guangxi [17, 18], respectively) have yielded contradictory conclusions.

To evaluate the effect of HLA-A and its neighboring gene on NPC susceptibility, we conducted a meta-analysis on the association between the five most frequently studied chromosome 6p21.3 SNPs (rs9260734, rs2517713, rs3129055, rs29232, and rs29230) and NPC susceptibility. Thus far, no meta-analysis has been conducted to explore the overall NPC risk and the genetic heterogeneity associated with chromosome 6p21 SNPs. In the current study, we postulated that moderate heterogeneity in rs29232 might contribute to the regional variation in NPC incidence rates.

## Methods

### Identification and eligibility of relevant studies

We reviewed the literature on PubMed for all relevant reports (the most recent search update was December 10, 2014), using the search terms “NPC,” “association,” and “HLA,” and limiting the results to English-language papers. In this meta-analysis, studies had to fulfill the following criteria: 1) evaluate the correlation between SNPs mapped by GWASs in the HLA-A region and NPC in Han Chinese populations, 2) use a case–control design, and 3) report the genotype frequency for both cases and controls and/or odds ratios (ORs).

### Description of studies

The meta-analysis was based on summary data reported by three previous GWASs on NPC [16–18] and four follow-up case–control studies [19–22] focusing on the HLA class I region. We extracted data according to the aforementioned inclusion criteria. The following data was collected from each study: 1) first author’s name, 2) year of publication, 3) sample collection area, 4) genotyping platform, 5) SNPs assessed, 6) number of cases and controls, 7) sex ratio, and 8) age range (Table 1). Twelve SNPs discovered by Tse *et al*. [16] were used as major targets for analysis; the citation of these SNPs was standardized using *rs* numbers, as suggested by Tse *et al*. For rs2517713, two SNPs (rs2860580 and rs9260475) acted as surrogates according to the strong LD relationship between the target and surrogate SNPs in the HapMap Han Chinese in Beijing (CHB) population (rs2860580–rs2517713: D’ = 1, *r*^*2*^ = 0.90; rs9260475–rs2517713: D’ = 1, *r*^*2*^ = 0.91). When available, the following information was obtained from the studies and included in the meta-analysis: 1) minor allele frequency (MAF) in the case and control samples, 2) *P* values for the original association, and 3) ORs and 95 % confidence intervals (CIs). When the target SNPs were genotyped in both the discovery and validation groups, the combined genotyping data was used.

**Table 1:** Characteristics of the studies included in the meta-analysis

		Stage I								Stage II						
	Sample	Genotyping		Sample		Gender (%)^*a*^		Age^*b*^		Genotyping	Sample		Gender (%)^*a*^		Age^*b*^	
Study	Population	Method	SNPs	Case	Control	Case	Control	Case	Control	Method	Case	Control	Case	Control	Case	Control
Tse (2009)	Taiwan	Illumina Hap550	480,365	277	285	76	67	49 (12)	50 (14)	TaqMan	635	1,640	73	73	50 (13)	59 (14)
Bei (2010)	Guangdong^*c*^	Illumina Hap610	464,328	1,583	1,894	73	69	46 (11)	47 (11)	TaqMan	3,507	3,063	74	67	46 (12)	44 (12)
Tang (2012)	Guangxi and Guangdong	Affymatrix 6.0	591,458	567	476	-	-	-	-	TaqMan	923	1,105	-	-	-	-
Li (2011)	Guangdong	TaqMan	233	360	360	72	34	46 (11)	41 (9)							
Zhao (2012)	Guangdong	SNPstream	100	206	180	71	67	-	-	Sequenome	535	525	73	76	-	-
Hsu (2012)	Taiwan	TaqMan	12	337	286	70	70	46	46							
Gao (2014)	Guangdong and Guangxi	TaqMan	16	350	619	67	43	45 (11)	46 (10)	TaqMan	816	1721	73	61	45 (11)	46 (12)

^*a*^Gender: Male percentage. ^*b*^Age: mean and standard deviation. ^*c*^Most of the samples were from Guangdong, except 922 GWAS controls were Han Chinese from Singapore

### Assessment of publication bias

Typical publication bias is a result of small sample sizes. Although the studies included in the meta-analysis had an adequate number of cases, the sample sizes were small compared with those of GWASs on other types of cancer. Furthermore, a low level of population admixture in a large study can cause publication bias. Therefore, potential publication bias was assessed using funnel and P–M plots [23, 24]. Funnel plotting and Egger’s linear regression test were performed using the Metafor package [23] in R [25], Version 3.0.2. When publication bias occurred, the funnel plot was noticeably asymmetric. Egger’s linear regression test was used to test the funnel-plot symmetry. The M values of P–M plots represented the posterior probability that an effect existed in each study. A low M value (<0.1) suggested that the study had no effect, and therefore, such studies were excluded from further analysis [24].

### Meta-analysis

All meta-analysis results presented in this report were calculated using the Metasoft software package, Version 2.0.1 [26]. The P–M plot, forest plot, and funnel plot were plotted using Metafor [27]. To evaluate the association between 6p21.3 SNPs and the risk of NPC, we calculated the pooled ORs and associated 95 % CIs. Standard meta-analysis involving the fixed effects model and conventional random effects model was conducted using the standard error and effect size reported in each study [24, 27]. If the target SNPs were genotyped in both discovery and validation stages (Table 1), the combined data was used, otherwise only stage I data was used for meta-analysis. The fixed effects model made a conditional inference on the heterogeneity among the true effects, whereas the conventional random effects model treated the heterogeneity as purely random.　Sensitivity analysis was conducted to assess the potential influences of any single study on the pooled ORs. In each meta-analysis, included studies were individually removed to ensure that no study significantly altered the pooled ORs and associated *P* values. Power analysis was conducted using the Power and Sample Size Calculation software Version 3.1.2 [28, 29].

### Test of heterogeneity

The heterogeneity effect was quantified using the I^2^ test [30]. The I^2^ values ranged from 0 to 100 %, and values of 25, 50, and 75 % were considered to represent low, moderate, and high levels of heterogeneity, respectively. Heterogeneity was estimated using Cochran’s Q statistic, and *P* < 0.1 was considered to indicate significant heterogeneity [31].

## Results

### Study selection

The primary search yielded 59 articles, of which seven were identified as potentially relevant, following a review of the title and abstract. In total, seven studies were included in the meta-analysis after a full-text review (Additional file 1: Figure S1). The overall study population in the current meta-analysis comprised 20,151 subjects, of which 8994 were patients with NPC and 11,157 were controls. We analyzed seven studies that examined Han Chinese subjects from Taiwan [16, 19], Guangdong [17, 18, 20–22], and Guangxi [18, 22] (Table 1) and assessed five chromosome 6p21.3 SNPs: (rs9260734 [*HCG6*], rs2517713 [*HLA-A*], rs3129055 [*HLA-F*], rs29232 [*GABBR1*], and rs29230 [*GABBR1*]). Two surrogate SNPs, rs2860580 [17] and rs9260475 [21], represented rs2517713 because the LD (D’ = 1, *r*^*2*^ > 0.9) between the target and surrogate SNPs in the HapMap CHB population was extremely high [32].

### Publication bias and synthesis of results

We used P–M and funnel plots to assess the publication bias of the included studies, detecting no evidence of potential publication bias in our target SNPs (Additional file 1: Figure S2). As expected, rs2517713 of the *HLA-A* gene was the most significantly associated with NPC (OR = 0.58, 95 % CI = 0.55–0.61, *P* = 1.08 × 10^−91^) (Table 2). Analysis of three of the five SNPs did not reveal heterogeneity (I^2^ = 0). Although we used surrogate SNPs (rs2860580 and rs9260475) in the meta-analysis of rs2517713, we did not observe publication bias (Additional file 1: Figure S2b) or heterogeneity (I^2^ = 0, Table 2), suggesting that SNPs with high LD (D’ = 1, *r*^*2*^ > 0.9) could be treated as the same SNP in the meta-analysis. We observed a moderate level of heterogeneity in rs3129055 (I^2^ = 58 %, *P* = 0.0361) and rs29232 (I^2^ = 47 %, *P* = 0.1091); however, the heterogeneity for rs29232 was not statistically significant (*P* > 0.1). Because Cochran’s Q statistic is severely underpowered in analyses with only four to five studies, heterogeneity might still exist despite a lack of nominal statistical significance [33]. Two SNPs that exhibited heterogeneity (rs3129055 and rs29232) were the same SNPs independent from the *HLA-A* effect [16]. The random effect of rs29232 exhibited a highly significant *P* value of 1.90 × 10^−16^ (OR = 1.47, 95 % CI = 1.34–1.61); however, the random effect of rs3129055 (*P =* 1.43 × 10^−5^, OR = 1.28, 95 % CI = 1.14–1.42) did not exhibit genome-wide significance (<10^−7^).

**Table 2:** Meta-analysis of the associations between chromosome 6p21 SNPs and NPC susceptibility

			Sample		MAF					Heterogeneity	
Gene	SNP	Study	Case	Control	Case	Control	*P*	OR	95 % CI	*I*^*2*^ %	*P*
*HCG9*	rs9260734	Tse (2009)	912	1,925	0.22	0.34	6.77E-18	0.54	0.47–0.62		
		Bei (2010)	1,583	1,894	0.22	0.33	9.80E-22	0.56	0.49–0.67		
		Tang (2012)	923	1,105	-	-	2.63E-11	0.59	0.50–0.69		
		Li (2011)	360	360	0.25	0.32	9.70E-03	0.74	0.53–1.01		
		Zhao (2012)	535	525	-	0.32	3.30E-07	0.60	0.49–0.73		
		Hsu (2012)	336	288	0.24	0.35	1.88E-03	0.57	0.40–0.81		
		Guo (2014)	1166	2340	-	-	5.96E-17	0.60	0.53–0.68		
		Fixed effect					9.68E-57	0.59	0.55–0.63	0	0.7774
		Random effect					9.68E-57	0.59	0.55–0.63		
*HLA-A*	rs2517713	Tse (2009)	912	1,925	0.24	0.38	3.90E-20	0.53	0.47–0.61		
	rs2860580^*a*^	Bei (2010)	5,090	4,957	-	-	3.65E-65	0.58	0.54–0.62		
		Tang (2012)	923	1,105	-	-	1.92E-11	0.60	0.52–0.70		
		Li (2011)	360	360	0.28	0.36	5.30E-04	0.67	0.48–0.91		
	rs9260475^*a*^	Zhao (2012)	206	180	-	0.38	1.00E-04	0.49	0.34–0.70		
		Hsu (2012)	337	286	0.26	0.38	1.90E-03	0.58	0.41–0.82		
		Guo (2014)	1166	2340	-	-	2.44E-16	0.62	0.55–0.70		
		Fixed effect					1.08E-91	0.58	0.55–0.61	0	0.5265
		Random effect					1.08E-91	0.58	0.55–0.61		
*HLA-F*	rs3129055	Tse (2009)	912	1,925	0.41	0.31	7.36E-11	1.51	1.34–1.71		
		Bei (2010)	1,583	1,894	0.37	0.30	3.00E-07	1.31	1.19–1.58		
		Tang (2012)	923	1,105	-	-	3.43E-02	1.17	1.01–1.34		
		Li (2011)	360	360	0.36	0.34	3.69E-01	1.11	0.81–1.50		
		Hsu (2012)	344	294	0.38	0.30	4.44E-02	1.42	1.02–1.98		
		Guo (2014)	1166	2340	-	-	2.00E-02	1.14	1.02–1.28		
		Fixed effect					6.44E-12	1.26	1.18–1.34	58	0.0361
		Random effect					1.43E-05	1.28	1.14–1.42		
*GABBR1*	rs29232	Tse (2009)	912	1,925	0.59	0.46	8.97E-17	1.67	1.48–1.88		
		Bei (2010)	1,583	1,894	0.53	0.43	3.90E-18	1.56	1.43–1.75		
		Tang (2012)	923	1,105	-	-	4.35E-06	1.36	1.20–1.56		
		Hsu (2012)	342	290	0.53	0.41	2.41E-03	1.63	1.19–2.24		
		Guo (2014)	1166	2340	-	-	1.85E-08	1.35	1.21–1.49		
		Fixed effect					1.46E-30	1.45	1.36–1.54	47	0.1092
		Random effect					1.90E-16	1.47	1.34–1.61		
*GABBR1*	rs29230	Tse (2009)	912	1,925	0.18	0.26	4.77E-09	0.64	0.56–0.75		
		Bei (2010)	1,583	1,894	0.19	0.27	1.30E-12	0.64	0.54–0.75		
		Tang (2012)	923	1,105	-	-	9.48E-09	0.61	0.52–0.72		
		Li (2011)	360	360	0.18	0.27	1.43E-04	0.61	0.42–0.86		
		Hsu (2012)	341	290	0.17	0.25	2.34E-02	0.63	0.43–0.93		
		Guo (2014)	1166	2340	-	-	1.36E-13	0.62	0.57–0.67		
		Fixed effect					5.78E-34	0.62	0.57–0.67	0	0.9988
		Random effect					5.78E-34	0.62	0.57–0.67		

^*a*^SNPs used as surrogate for rs2517713. The *r*^*2*^ between rs2860580 and rs2517713 was 0.91; *r*^*2*^ between rs2860580 and rs9260475 was 0.90

### Sensitivity analysis

To investigate the potential influence of a single study on the overall meta-analysis estimation, we omitted one study at a time. Similar results were obtained for the three SNPs that did not exhibit heterogeneity (rs9260734, rs2517713, and rs29230) regardless of any study being omitted (Additional file 1: Table S1), indicating that our results were supported by reliable data. The ORs and I^2^ of the two SNPs exhibiting heterogeneity (rs3129055 and rs29232) calculated using the sensitivity test differed. Power analysis of each target SNP in the total sample size involved the following assumptions: two-tailed α = 0.05 and the frequency of minor alleles in control samples. According to the ORs obtained through meta-analysis, the present sample size showed a power of 1.00 for detecting a significant association.

### Subgroup analysis

We stratified the studied population on the basis of region, namely the moderate-incidence region (Taiwan) [16, 19] and high-incidence regions (Guangdong and Guangxi) [17, 18, 22] (Table 3). The heterogeneity decreased markedly from 47 to 0 % in both the moderate- and high-incidence regions, suggesting that the heterogeneity of rs29232 was attributable to the geographical difference in NPC incidence.

**Table 3:** Meta-analysis of rs29232 in studies with samples from various incidence regions

		Sample		Fixed effect			Random effect			Heterogeneity	
	Population	Case	Control	*P*	OR	95 % CI	*P*	OR	95 % CI	*I*^*2*^ %	*P*
All		4,926	7,554	1.46E-30	1.45	1.36–1.54	1.90E-16	1.51	1.34–1.61	47	0.1092
Moderate incidence region	Taiwan	1,254	2,215	3.43E-13	1.70	1.47–1.95	3.43E-13	1.70	1.47–1.95	0	0.7998
High incidence region	Guangdong and Guangxi^*a*^	3,672	5,339	2.92E-20	1.40	1.30–1.49	2.92E-13	1.40	1.30–1.49	0	0.4627

^*a*^Most of the samples were from high incidence region (Guangdong and Guangxi) except 922 controls were Han Chinese from Singapore

## Discussion and conclusions

In the present meta-analysis, four of the five chromosome 6p21.3 polymorphisms exhibited strong and consistent positive associations with NPC. The three SNPs (rs2517713, rs9260734, and rs29230) yielded highly consistent results with no heterogeneity, despite differences in the genotyping platform (Table 1) and the use of surrogate SNPs for rs2517713 (Table 2). The strongest association was discovered in rs2517713 near the *HLA-A* gene (*P* = 1.08 × 10^−91^, OR = 0.58, 95 % CI = 0.55–0.61), further confirming the critical role of the HLA-A gene in the susceptibility of NPC.

NPC has a distinct ethnic and geographic distribution. The allele frequencies of NPC-associated alleles (HLA-A*1101, HLA-A*0207, and HLA-B*5801) in the human genome are consistent with the geographical distribution of NPC incidence in southeastern Asia [10–12], suggesting that the genetic differences among populations might play a crucial role in NPC incidence. However, in the NPC association studies, the frequency of HLA alleles did not differ noticeably in populations with dissimilar incidence rates, suggesting that HLA genes might not directly cause this difference. In the current meta-analysis, we did not observe heterogeneity in the *HLA-A* SNPs, which supports our hypothesis. By contrast, our analysis indicated that rs29232 exhibited distinct features in Han Chinese populations in regions with different incidence rates. First, the heterogeneity of rs29232 was markedly reduced when we stratified the meta-analysis according to the incidence region. Second, in the subgroup analysis, the OR of rs29232 was higher in moderate-incidence regions than in the high-incidence regions. Finally, in previous NPC association studies, rs29232 was independent of the *HLA-A* effect in moderate-incidence regions, but not in high-incidence regions. These results suggest that rs29232 might contribute to the difference in NPC incidence in Han Chinese populations.

Studies conducted in regions with different incidence rates have yielded inconsistent results regarding rs29232. A GWAS conducted in Taiwan, a moderate-NPC-incidence region, using multiple logistic regression analysis and stepwise logistic regression concluded that rs29232 was significantly associated with NPC, even after the removal of the *HLA-A* SNP (rs2517713) and sequence-based *HLA-A* alleles (HLA-A*0207/0215 N and HLA-A*110101/0121 N) [16]. Another independent post-GWAS case–control study conducted in Taiwan yielded similar results, indicating that *HLA-A* and rs29232 are likely to be independent risk factors for NPC, and that NPC risk is highest among people carrying homozygous HLA-A*0207 and rs29232 risk alleles [19]. By contrast, a GWAS conducted in Guangdong, a high-NPC-incidence region, revealed that the strength of the association with rs29232 greatly diminished after the researchers controlled for the effect of rs2860580 (*HLA-A*), whereas the strength of the association with rs2860580 (*HLA-A*) decreased after they adjusted for rs29232. Collectively, these results suggest that the associations with rs29232 and rs2860580 (*HLA-A*) were correlated rather than probabilistically independent [17]. In an NPC GWAS conducted in Guangdong and Guangxi, a similar multivariate logistic regression analysis indicated that rs29232- and *GABBR1*-related SNPs were nonsignificant after adjustment for *HLA-A*-related SNPs and alleles. The authors of the study stated that all the other significant associations identified were only proxies for HLA-A*1101 because of the strong LD within the region [18]. To summarize, study findings from moderate-incidence regions support the independent role of rs29232, whereas those from high-incidence regions indicate that only one true association signal (*HLA-A*) exists within this chromosome region.

In the current study, no heterogeneity was observed among studies except in rs3129055 (I^2^ = 58 %, *P* = 0.0361, *HLA-F*) and rs29232 (I^2^ = 47 %, *P* = 0.1092, *GABBR1*). However, the heterogeneity in rs29232 was not statistically significant (*P* > 0.1). Because tests of heterogeneity are severely underpowered in analyses of only a few studies [33], heterogeneity might still exist despite a lack of statistical significance. Since the meta-analysis result for rs3129055 did not achieve genome-wide significance (<10^−7^), we excluded it from further analysis. The moderate level of heterogeneity (I^2^ = 47) of rs29232 markedly decreased (I^2^ = 0) when we stratified the study population according to geographic region (Table 3), suggesting that regional differences in NPC incidence caused the heterogeneity. Furthermore, the ORs were higher in the moderate-incidence regions (OR = 1.70, 95 % CI = 1.47–1.95) than in the high-incidence regions (OR = 1.40, 95 % CI = 1.30–1.49). Since rs29232 was a significant association signal independent of the effect of the *HLA-A* gene in the moderate-incidence regions, we suspected that rs29232 caused the regional difference in NPC incidence among Han Chinese people. Compared with high-incidence populations, moderate-incidence populations might be more strongly affected by rs29232.

Although the current evidence on the role of rs29232 and the *GABBR1* gene seems self-contradictory, previous studies have provided consistent findings regarding the role of *GABBR1* in NPC. *GABBR1* encodes the protein gamma-aminobutyric acid B receptor 1 (GABBR1), a G protein-coupled receptor that forms a heterodimer with GABAB receptor 2, thereby triggering downstream signaling events involved in the proliferation, differentiation, and migration of cancer cells. One study reported that the intensity of the GABBR1 signal in tumor cells was significantly higher than that detected in adjacent normal epithelial cells (*P* < 0.001) in the immunohistochemical staining of NPC tissues [16].

The rs29232 SNP exhibited a lower LD (*r*^*2*^ < 0.6) than did all other SNPs in the HapMap CHB data set in the MHC region. This finding suggests that the role of rs29232 might not directly relate to the downstream *GABBR1* gene or other neighboring genes, but rather affect genes located in other regions or chromosomes through long-range *cis-*acting or *trans*-acting. Nevertheless a recent genome-wide SNP–SNP interaction analysis detected no significant interaction between rs29232 and other SNPs [34]. Further investigation of the role of rs29232 and its relationship with the incidence and etiology of NPC is vital. In explorations of the genetic factors underlying disease susceptibility, epidemiological factors, such as disease incidence rates, are rarely considered. In the current study, an association analysis of rs29232 in regions with high or moderate incidence rates was performed. Future studies conducted in low-incidence regions such as northern China may clarify the relationship between genetic factors and geographic incidence rates. In addition, using imputation or next-generation sequencing to explore the detailed genotypes near rs29232 can further reveal the genetic causes underlying the variation in regional NPC incidence rates. The current study suggested that genetic effects might be involved in epidemiological factors such as regional disease incidences. However, epidemiological factors have rarely been considered in large cross-region association studies. Thus, we suggest that future large cross-region meta-analyses include geographic incidence rates as potential confounding factors.

In this meta-analysis, the results indicated a strong effect of HLA-A on NPC susceptibility and a potential role of rs29232 in the regional differences in NPC incidence among Han Chinese people. However, this study had several limitations. First, although the conclusion was based on several previous studies, the heterogeneity for rs29232 observed in this study was nonsignificant. Second, although the number of subjects in the studies included in the analysis was high, the number of studies included was relatively low. Finally, meta-analyses constitute retrospective research and, thus, are subject to methodological limitations. To minimize potential bias, we used standard methods for study selection, data extraction, and data analysis. Nonetheless, the results presented here should be interpreted with caution until additional studies on rs29232 that control for incidence rates according to region are conducted.

## Additional file

Additional file 1: **Supplementary Table S1.** Sensitivity analysis of meta-analysis. **Supplementary Figure S1**. PRISMA Flow chart of literature review and study selection. **Supplementary Figure S2**. Forest plots, funnel plots and P-M plots for NPC association. (PDF 1068 kb)

## Abbreviations

NPC: Nasopharyngeal carcinoma
SNP: Single-nucleotide polymorphism
HLA: Human leukocyte antigen
GWAS: Genome-wide association study
LD: Linkage disequilibrium
MAF: Minor allele frequency
OR: Odds ratio
CI: Confidence interval
FE: Fixed effect
RE: Random effect
CHB: Han Chinese in Beijing
GABBR1: Gamma-aminobutyric acid B receptor 1
