# Supplementary material for: Heterogeneity revealed through meta-analysis might link geographical differences with nasopharyngeal carcinoma incidence in Han Chinese populations
Source: BMC Cancer. 2015 Aug 26;15:598. doi: 10.1186/s12885-015-1607-0 (PMC4549009; doi:10.1186/s12885-015-1607-0)
Supplement: Additional file 1: — Supplementary Table S1. Sensitivity analysis of meta-analysis. Supplementary Figure S1. PRISMA Flow chart of literature review and study selection. Supplementary Figure S2. Forest plots, funnel plots and P-M plots for NPC association. (PDF 1068 kb) [file 12885_2015_1607_MOESM1_ESM.pdf]

Supplementary Table S1 Sensitivity analysis of meta-analysis

SNP	Study Omitted	Fixed Effect		Random Effect		Heterogeneity	
		OR (95% CI)	P[a]	OR (95% CI)	P[a]	I^2 (%)	P[b]
rs9260734	Tse (2009)	0.60 (0.56-0.64)	5.18E-47	0.60 (0.56-0.64)	5.18E-47	0	0.8682
	Bei (2010)	0.59 (0.55-0.63)	1.28E-45	0.59 (0.55-0.63)	1.28E-45	0	0.6812
	Tang (2012)	0.59 (0.55-0.63)	3.15E-48	0.59 (0.55-0.63)	3.15E-48	0	0.6629
	Li (2011)	0.58 (0.54-0.62)	2.38E-56	0.58 (0.54-0.62)	2.38E-56	0	0.9201
	Zhao (2012)	0.59 (0.55-0.63)	5.71E-51	0.59 (0.55-0.63)	5.71E-51	0	0.6693
	Hsu (2012)	0.59 (0.55-0.63)	1.43E-54	0.59 (0.55-0.63)	1.43E-54	0	0.6668
	Guo(2014)	0.58 (0.54-0.63)	1.49E-39	0.58 (0.54-0.63)	1.49E-39	0	0.6908
		**0.59 (0.55-0.63)**	**9.68E-57**	**0.59 (0.55-0.63)**	**9.68E-57**	**0**	**0.7774**
rs2517713	Tse (2009)	0.59 (0.56-0.62)	4.21E-80	0.59 (0.56-0.62)	4.21E-80	0	0.7783
	Bei (2010)	0.59 (0.54-0.63)	4.62E-43	0.59 (0.54-0.63)	7.18E-42	2	0.4052
	Tang (2012)	0.58 (0.55-0.61)	4.48E-81	0.58 (0.55-0.61)	4.48E-81	0	0.4206
	Li (2011)	0.58 (0.55-0.61)	2.04E-90	0.58 (0.55-0.61)	2.04E-90	0	0.4823
	Zhao (2012)	0.59 (0.56-0.62)	1.49E-88	0.59 (0.56-0.62)	1.49E-88	0	0.5186
	Hsu (2012)	0.58 (0.55-0.61)	1.38E-89	0.58 (0.55-0.62)	1.39E-83	3	0.3995
	Guo(2014)	0.58 (0.54-0.61)	2.41E-79	0.58 (0.54-0.61)	2.41E-79	0	0.5643
		**0.58 (0.55-0.61)**	**1.08E-91**	**0.58 (0.55-0.61)**	**1.08E-91**	**0**	**0.5265**
rs3129055	Tse (2009)	1.21 (1.13-1.30)	1.16E-07	1.22 (1.11-1.34)	1.83E-05	29	0.2308
	Bei (2010)	1.23 (1.14-1.32)	4.78E-08	1.25 (1.10-1.43)	7.56E-04	60	0.0398
	Tang (2012)	1.28 (1.19-1.38)	3.35E-11	1.30 (1.14-1.49)	1.07E-04	63	0.0295
	Li (2011)	1.27 (1.18-1.35)	5.42E-12	1.29 (1.15-1.46)	2.18E-05	64	0.0247
	Hsu (2012)	1.25 (1.17-1.34)	4.45E-11	1.26 (1.12-1.42)	1.25E-04	65	0.0229
	Guo(2014)	1.32 (1.22-1.44)	9.54E-12	1.32 (1.17-01.49)	3.34E-06	46	0.1189
		**1.26 (1.18-1.34)**	**6.44E-12**	**1.28 (1.14-1.42)**	**1.43E-05**	**58**	**0.0361**
rs29232	Tse (2009)	1.40 (1.31-1.50)	4.31E-22	1.40 (1.31-1.50)	4.31E-22	0	0.4771
	Bei (2010)	1.43 (1.33-1.54)	4.60E-23	1.47 (1.30-1.66)	4.78E-10	59	0.0644
	Tang (2012)	1.48 (1.37-1.59)	9.27E-26	1.51 (1.34-1.69)	2.08E-12	52	0.0977
	Hsu (2012)	1.44 (1.35-1.53)	1.17E-28	1.46 (1.32-1.61)	1.16E-13	57	0.0732
	Guo(2014)	1.50 (1.39-1.62)	9.64E-25	1.51 (1.36-1.69)	4.98E-14	43	0.1533
		**1.45 (1.36-1.54)**	**1.46E-30**	**1.47 (1.34-1.61)**	**1.90E-16**	**47**	**0.1092**
rs29230	Tse (2009)	0.62 (0.57-0.67)	2.37E-29	0.62 (0.57-0.67)	2.37E-29	0	0.9953
	Bei (2010)	0.61 (0.56-0.67)	2.23E-27	0.61 (0.56-0.67)	2.23E-27	0	0.9988
	Tang (2012)	0.62 (0.57-0.68)	6.49E-26	0.62 (0.57-0.68)	6.49E-26	0	0.9960
	Li (2011)	0.62 (0.57-0.67)	2.64E-32	0.62 (0.57-0.67)	2.64E-32	0	0.9947
	Hsu (2012)	0.62 (0.57-0.67)	8.46E-33	0.62 (0.57-0.67)	8.46E-33	0	0.9947
	Guo(2014)	0.62 (0.57-0.68)	9.35E-24	0.62 (0.57-0.68)	9.35E-24	0	0.9968
		**0.62 (0.57-0.67)**	**5.78E-34**	**0.62 (0.57-0.67)**	**5.78E-34**	**0**	**0.9988**

OR, Odds ratio; CI, Confidence Interval; [a]Meta analysis P-value in the remaining sample; [b]Heterogeneity test P-value in the remaining sample;

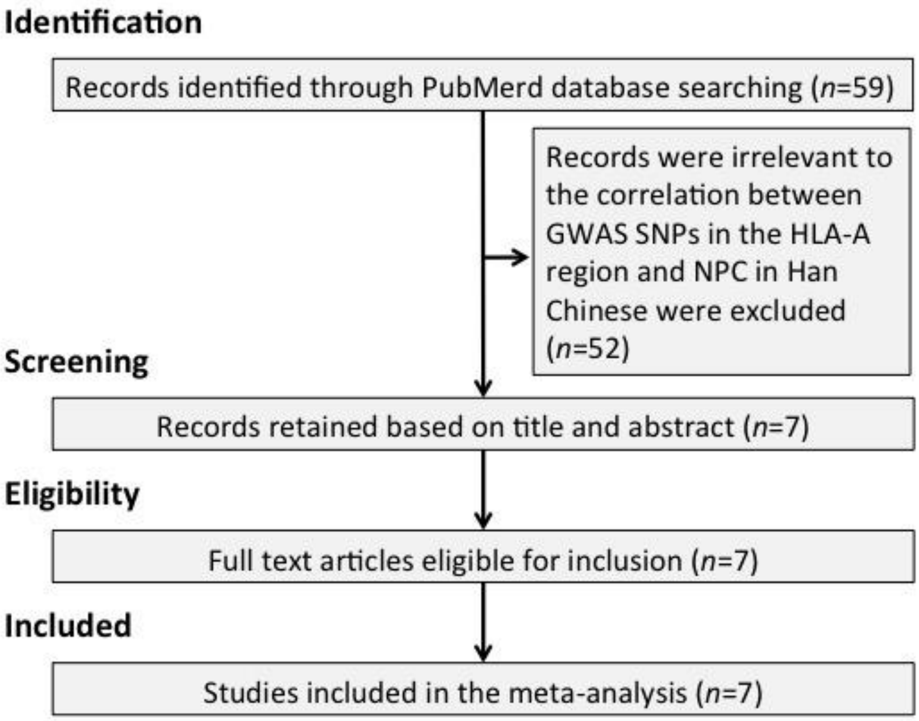

Supplementary Figure S1 PRISMA Flow chart of literature review and study selection.

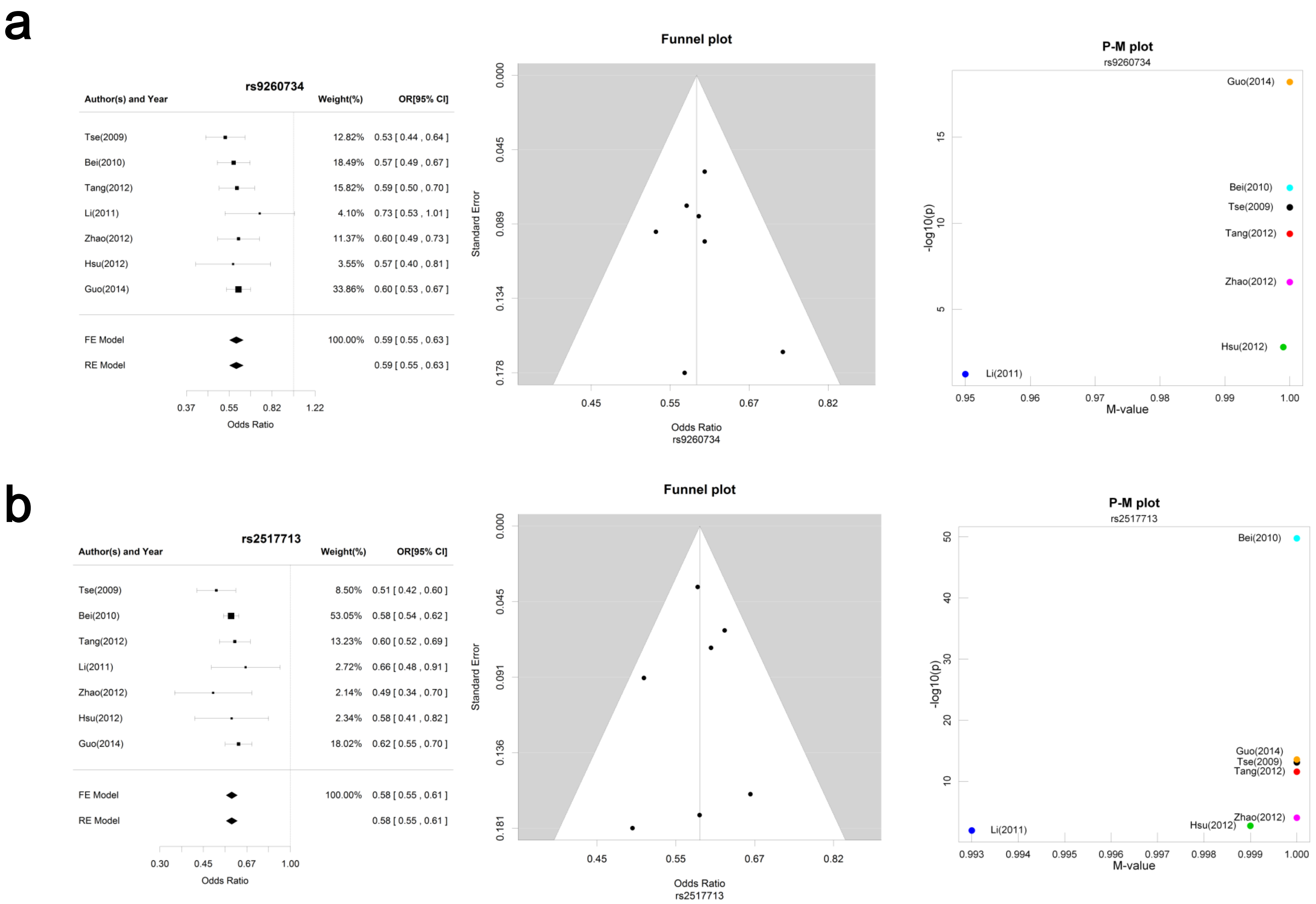

Supplementary Figure S2 Forest plots, funnel plots and P-M plots for NPC association (part I)

c

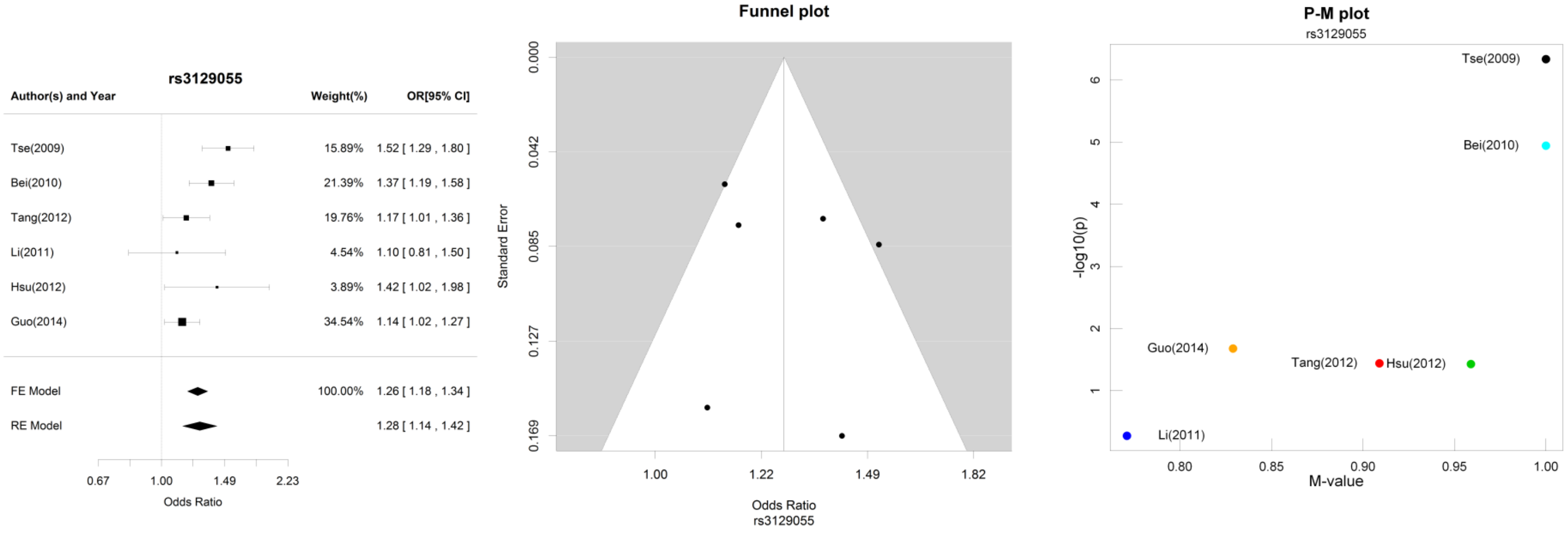

d

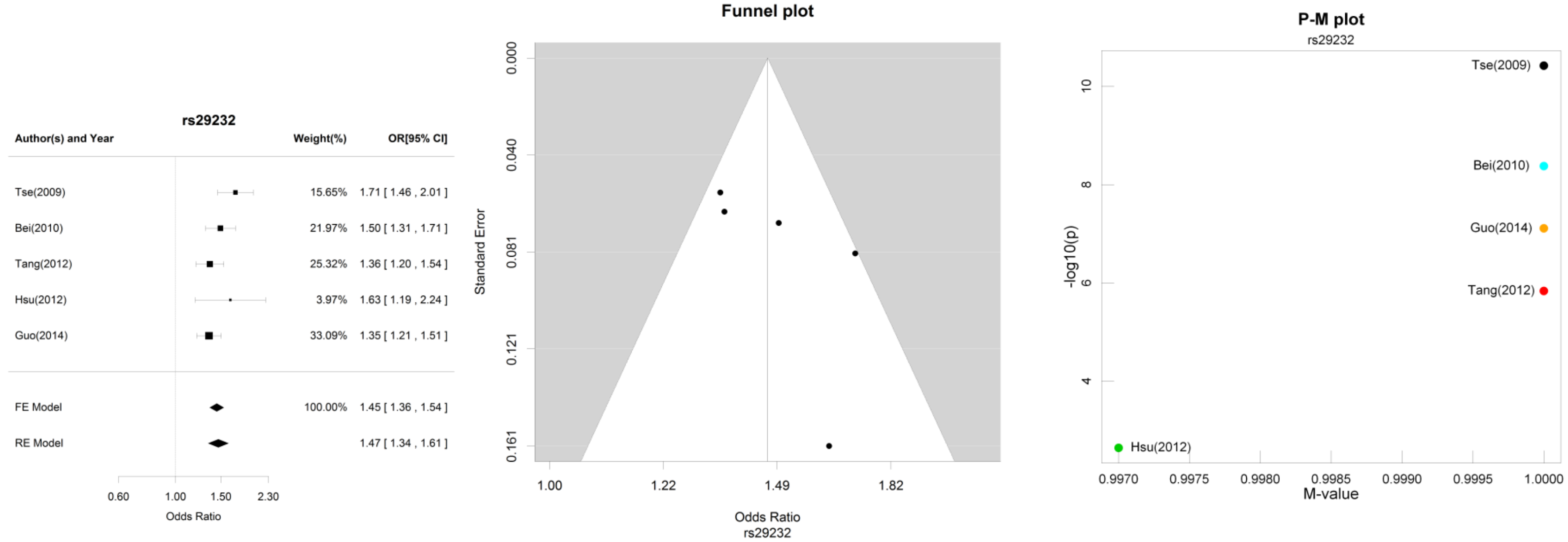

Supplementary Figure S2 Forest plots, funnel plots and P-M plots for NPC association (part II).

e

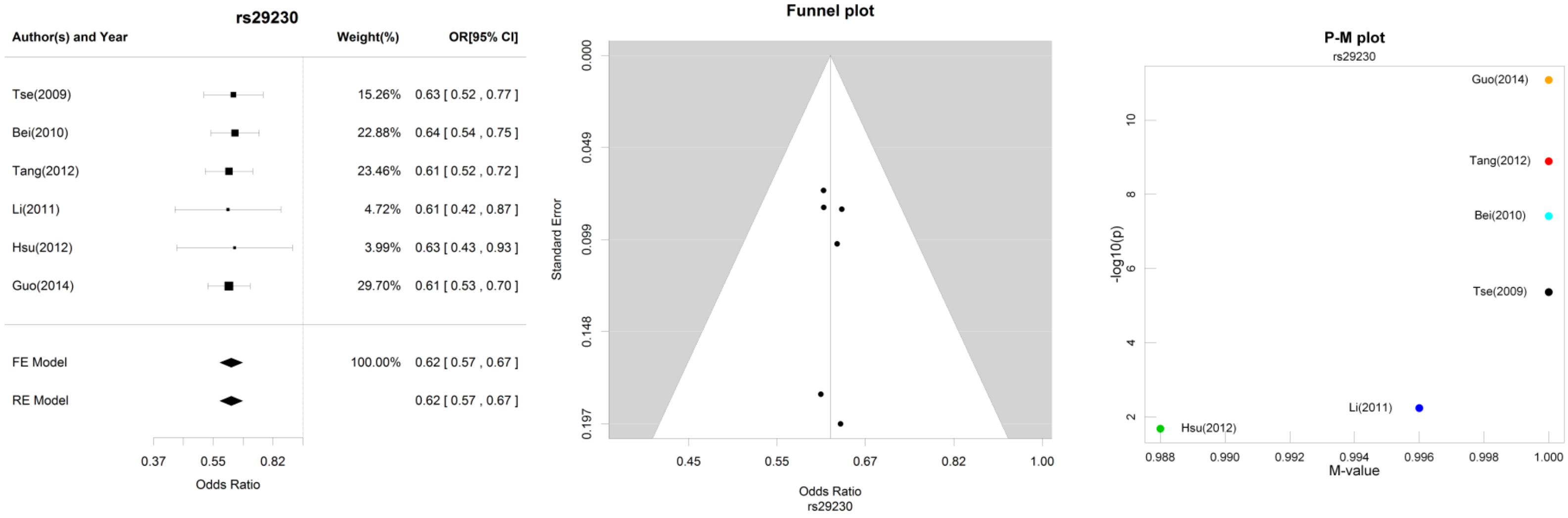

Supplementary Figure S2 Forest plots, funnel plots and P-M plots for NPC association (part III). **a)** rs9260734; **b)** rs2517713; **c)** rs3129055; **d)** rs29232; **e)** rs29230. **Left**: Forest plots of ORs with 95% CIs for polymorphisms and NPC susceptibility. **Middle** and **Right**: funnel plots and P–M plots represent the publication bias test.

PRISMA 2009 Checklist

Section/topic	#	Checklist item	Heading
**TITLE**			
Title	1	Identify the report as a systematic review, meta-analysis, or both.	Title
**ABSTRACT**			
Structured summary	2	Provide a structured summary including, as applicable: background; objectives; data sources; study eligibility criteria, participants, and interventions; study appraisal and synthesis methods; results; limitations; conclusions and implications of key findings; systematic review registration number.	Abstract
**INTRODUCTION**			
Rationale	3	Describe the rationale for the review in the context of what is already known.	Introduction
Objectives	4	Provide an explicit statement of questions being addressed with reference to participants, interventions, comparisons, outcomes, and study design (PICOS).	Introduction
**METHODS**			
Protocol and registration	5	Indicate if a review protocol exists, if and where it can be accessed (e.g., Web address), and, if available, provide registration information including registration number.	N/A
Eligibility criteria	6	Specify study characteristics (e.g., PICOS, length of follow-up) and report characteristics (e.g., years considered, language, publication status) used as criteria for eligibility, giving rationale.	Identification and eligibility of relevant studies
Information sources	7	Describe all information sources (e.g., databases with dates of coverage, contact with study authors to identify additional studies) in the search and date last searched.	Identification and eligibility of relevant studies
Search	8	Present full electronic search strategy for at least one database, including any limits used, such that it could be repeated.	Identification and eligibility of relevant studies
Study selection	9	State the process for selecting studies (i.e., screening, eligibility, included in systematic review, and, if applicable, included in the meta-analysis).	Identification and eligibility of relevant studies
Data collection process	10	Describe method of data extraction from reports (e.g., piloted forms, independently, in duplicate) and any processes for obtaining and confirming data from investigators.	Description of studies
Data items	11	List and define all variables for which data were sought (e.g., PICOS, funding sources) and any assumptions and simplifications made.	Description of studies
Risk of bias in individual studies	12	Describe methods used for assessing risk of bias of individual studies (including specification of whether this was done at the study or outcome level), and how this information is to be used in any data synthesis.	Assessment of publication bias
Summary measures	13	State the principal summary measures (e.g., risk ratio, difference in means).	Meta-analysis

PRISMA 2009 Checklist

Section/topic	#	Checklist item	Reported on page #
Synthesis of results	14	Describe the methods of handling data and combining results of studies, if done, including measures of consistency (e.g., I^2) for each meta-analysis.	Test of heterogeneity
Risk of bias across studies	15	Specify any assessment of risk of bias that may affect the cumulative evidence (e.g., publication bias, selective reporting within studies).	Assessment of publication bias
Additional analyses	16	Describe methods of additional analyses (e.g., sensitivity or subgroup analyses, meta-regression), if done, indicating which were pre-specified.	Meta-analysis
**RESULTS**			
Study selection	17	Give numbers of studies screened, assessed for eligibility, and included in the review, with reasons for exclusions at each stage, ideally with a flow diagram.	Results
Study characteristics	18	For each study, present characteristics for which data were extracted (e.g., study size, PICOS, follow-up period) and provide the citations.	Results
Risk of bias within studies	19	Present data on risk of bias of each study and, if available, any outcome level assessment (see item 12).	Results
Results of individual studies	20	For all outcomes considered (benefits or harms), present, for each study: (a) simple summary data for each intervention group (b) effect estimates and confidence intervals, ideally with a forest plot.	Results
Synthesis of results	21	Present results of each meta-analysis done, including confidence intervals and measures of consistency.	Results
Risk of bias across studies	22	Present results of any assessment of risk of bias across studies (see Item 15).	Results
Additional analysis	23	Give results of additional analyses, if done (e.g., sensitivity or subgroup analyses, meta-regression [see Item 16]).	Results
**DISCUSSION**			
Summary of evidence	24	Summarize the main findings including the strength of evidence for each main outcome; consider their relevance to key groups (e.g., healthcare providers, users, and policy makers).	Discussion
Limitations	25	Discuss limitations at study and outcome level (e.g., risk of bias), and at review-level (e.g., incomplete retrieval of identified research, reporting bias).	Discussion
Conclusions	26	Provide a general interpretation of the results in the context of other evidence, and implications for future research.	Discussion
**FUNDING**			
Funding	27	Describe sources of funding for the systematic review and other support (e.g., supply of data); role of funders for the systematic review.	Acknowledgements

From: Moher D, Liberati A, Tetzlaff J, Altman DG, The PRISMA Group (2009). Preferred Reporting Items for Systematic Reviews and Meta-Analyses: The PRISMA Statement. PLoS Med 6(6): e1000097. doi:10.1371/journal.pmed1000097

For more information, visit: **www.prisma-statement.org**.
